# The effectiveness of a mHealth-based integrated hospital-community-home program for people with type 2 diabetes in transitional care: a protocol for a multicenter pragmatic randomized controlled trial

**DOI:** 10.1186/s12875-022-01814-8

**Published:** 2022-08-05

**Authors:** Wenhui Zhang, Pei Yang, Hongyan Wang, Xinxin Pan, Yanmei Wang

**Affiliations:** 1 Nursing Department, Gongli Hospital, Pudong, Shanghai China; 2 The School of Nursing, Ningxia Medical University, Yinchuan, Ningxia China

**Keywords:** Type 2 diabetes, Transitional care, Mobile health, Smartphone applications, Apps, Self-management

## Abstract

**Background:**

Diabetes is a progressive condition requiring long-term medical care and self-management. The ineffective transition from hospital to community or home health care may result in poor glycemic control and increase the risk of serious diabetes-related complications. In China, the most common transitional care model is home visits or telephone interventions led by a single healthcare setting, with a lack of cooperation between specialists and primary care, which leads to inadequate service and discontinuous care. Thus, an integrated hospital-community-home (i-HCH) transitional care program was developed to promote hospital and community cooperation and provide comprehensive and continuous medical care for type 2 diabetes mellitus (T2DM) via mobile health (mHealth) technology.

**Methods:**

This protocol is for a multicenter randomized controlled trial in T2DM patients. Hospitalized patients diagnosed with T2DM who meet the eligibility criteria will be recruited. The patients will be randomly allocated to either the intervention or the control group and receive the i-HCH transitional care or usual transitional care intervention. The change in glycated hemoglobin is the primary outcome. Secondary outcome measures are blood pressure, lipids (total cholesterol, triglycerides, low-density lipoprotein, high-density lipoprotein), body mass index, self-management skills, quality of life, diabetes knowledge, transitional care satisfaction and the rate of readmission. The follow-up period of this study is six months.

**Discussion:**

The study will enhance the cooperation between local hospitals and communities for diabetes transitional care. Research on the effectiveness of diabetes outcomes will have potentially significant implications for chronic disease patients, family members, health caregivers and policymakers.

**Trial registration:**

Chinese Clinical Trial Registry ChiCTR1900023861: June 15, 2019.

## Background

Type 2 diabetes mellitus (T2DM) is a chronic metabolic disease that threatens human health and creates a large economic burden [1]. The ever-increasing prevalence of T2DM poses a severe burden to the health system [2, 3]. In China, T2DM affects 161.4 million people and contributes to 823,800 deaths. Diabetes-related costs reached 10.9 million USD in 2019, and they are expected to continue to grow [4]. Despite the health system's dedication to accelerating the development of chronic healthcare services, sufficient access for T2DM patients to health care and education has not been established [5, 6]. Diabetes awareness (36.5%), treatment (32.2%) and control (49.2%) rates are all at relatively low levels in China [7].

T2DM is a progressive condition that requires ongoing contact with medical settings and posthospital self-management to control blood glucose and reduce the risk of macro-microvascular complications and mortality [8, 9]. As inpatients transition from the hospital to the community and into the home, the increasing need for out-of-hospital patient care is crucial for healthcare organizations [10, 11]. Transitional care is generally recognized as an important aspect to ensure the quality of health care services and is widely used in chronic disease management. Transitional care incorporates a broad range of services designed to provide seamless care for patients as they move from one setting to another, and it has been proven to be effective in reducing unplanned admissions and mortality and improving care satisfaction [12–15].

Transitional care models are diverse in their health resources and local cultural characteristics [5]; they can be offered by teams or individuals [16, 17], include home visits or telephone coaching [18, 19], and be provided by the community or the hospital [20, 21]. The different forms have shown mixed results in terms of disease outcomes. The standard transitional care models for Chinese T2DM patients are relatively homogeneous and include home visits, telephone coaching and outpatient clinic [22, 23]. Patients passively obtain health information and lack access to interaction with their caregivers. And the content of health services is also limited in terms of health education, lacking methods and motivation for self-management and lifestyle change. In addition, T2DM patients need to travel between the hospital and the community for long-term medical services [24]. With separate operations in hospitals and communities in China, the transitional care team is usually led by a single care setting, leading to the interruption of services and health information [25]. A lack of solid collaboration between specialists and primary care introduces the potential for poor outcomes [15]. It is necessary to explore diverse access to health services and stratified care models to achieve continuous management, relationships, and information and effectively use community and medical resources.

Mobile health (mHealth), which refers to the delivery of health services via mobile devices such as wearable technology, smartphone applications (apps) and hand-held devices, has been rapidly developed and applied to numerous areas [26]. China has 986 million mobile internet users and a 70.4% internet penetration rate [27], and the vast internet market has increased the potential of mHealth to have considerable practical application for T2DM. Apps represent a valuable adjunct to provide low-cost and real-time ways to enable remote clinicians and personalized disease management and health information sharing [28]. The effectiveness and feasibility of app-related health care programs in T2DM management have been promising. These programs benefit self-management and quality of life in discharged patients with T2DM, aspects that should be promoted in community settings [29–33].

We designed a mHealth-based transitional care intervention that creates a support network with multiple agencies to establish a communication bridge between hospitals, communities and home healthcare providers. A professional diabetes-related app was developed to support a transitional care team providing continuous and remote health services for discharged patients. This paper describes the intervention protocol of a multicenter pragmatic randomized controlled trial (RCT) and hypothesizes that it will promote T2DM patients' glycemic control, self-management skills and other health outcomes. Our primary outcome is the change in glycated hemoglobin (HbA_1c_) at the 6-month follow-up; other outcomes are described below.

## Methods

### Study objective

The primary goal of this study is to assess whether the mHealth-based i-HCH transitional care program, by facilitating hospital-community collaboration and ensuring that patients receive continuity of care in the transition from hospital to the community or home with the use of mHealth technology, reduces T2DM patients' HbA_1c_ values compared to usual transitional care. The secondary goals are to (1) remotely monitor and dynamically assess risk factors for T2DM patients, implement personalized health interventions and education for patients to change poor health behaviors, improve patients' self-management skills and disease knowledge, and improve patients' lipid, blood pressure and body mass index (BMI) profiles; (2) through comprehensive interventions resulting in the overall improvement in patient quality of life, satisfaction with care, and reduction in patient readmissions; and (3) to understand the feasibility and applicability of the alliance between specialists in primary care in the clinical environment.

### Study design

This study is a multicenter pragmatic RCT with a 6-month follow-up period performed at a large general hospital and in 8 communities in Shanghai, China. The study will be conducted with a transitional care team that includes clinical nurses certified in diabetes care, physicians, endocrinologists, community nurses (CNs), general practitioners (GPs), pharmacists and administrative support staff to begin mHealth-based integrated hospital-community-home (i-HCH) transitional care to facilitate the glycemic control of discharged patients with T2DM and guide their self-management skills. Patients who meet the eligibility criteria will be included and randomized to receive the i-HCH transitional care or usual transitional care intervention. The allocation ratio will be 1:1. Due to the characteristics of the study, trial team members and participants will not be blinded, while the data collectors and statistical analysis assistants will be blinded.

### Participant recruitment and randomization

Patients admitted to the Department of Endocrinology in a large general hospital will be screened for eligibility based on electronic medical record data. The study eligibility criteria are as follows: (1) the participant is aged between 18 and 70 years old; (2) the participants' most recent HbA_1c_ ≥ 7.0% (53 mmol/mol); (3) the participant can use smartphones and apps; (4) the participant will return home and reside around the eight target communities; and (5) the participant can communicate and read in Chinese. The ineligibility criteria are as follows: (1) having type 1 or gestational diabetes; (2) lacking reliable internet access (no access to 4G,5G, or Wi-Fi connection); (3) being homeless or living in other care centers; (4) having debilitating complex medical conditions, e.g., end-stage cancer, dialysis, severe mental illness; and (5) refusal to sign an informed consent form or fall by the wayside.

Clinical nurses will explain the objective and procedure of the study face-to-face and give candidates a program booklet. If the patient participates, they will be formally referred to the study and provided informed consent. Then, the patients will be allocated to the intervention or control group using a random number generator programmed in SPSS (IBM Corp, version 23.0, Chicago, IL, USA).

### Study outcomes

The change in HbA_1c_ is the primary outcome. The secondary outcome measures are as follows: (1) clinical outcomes, including blood pressure, lipids (total cholesterol [TC], triglycerides [TGs], low-density lipoprotein [LDL], high-density lipoprotein [HDL]), and body mass index (BMI); (2) diabetes self-management skills assessed using the Summary of Diabetes Self-care Activities questionnaire (SDSCA) [34]; (3) quality of life using the Diabetes-Specific Quality of Life scale (DSQL) [35]; (4) diabetes knowledge using the Diabetes Knowledge Test (DKT) [36]; (5) transitional care satisfaction; and (6) the rate of readmission.

### Data collection

**Fig. 1:**
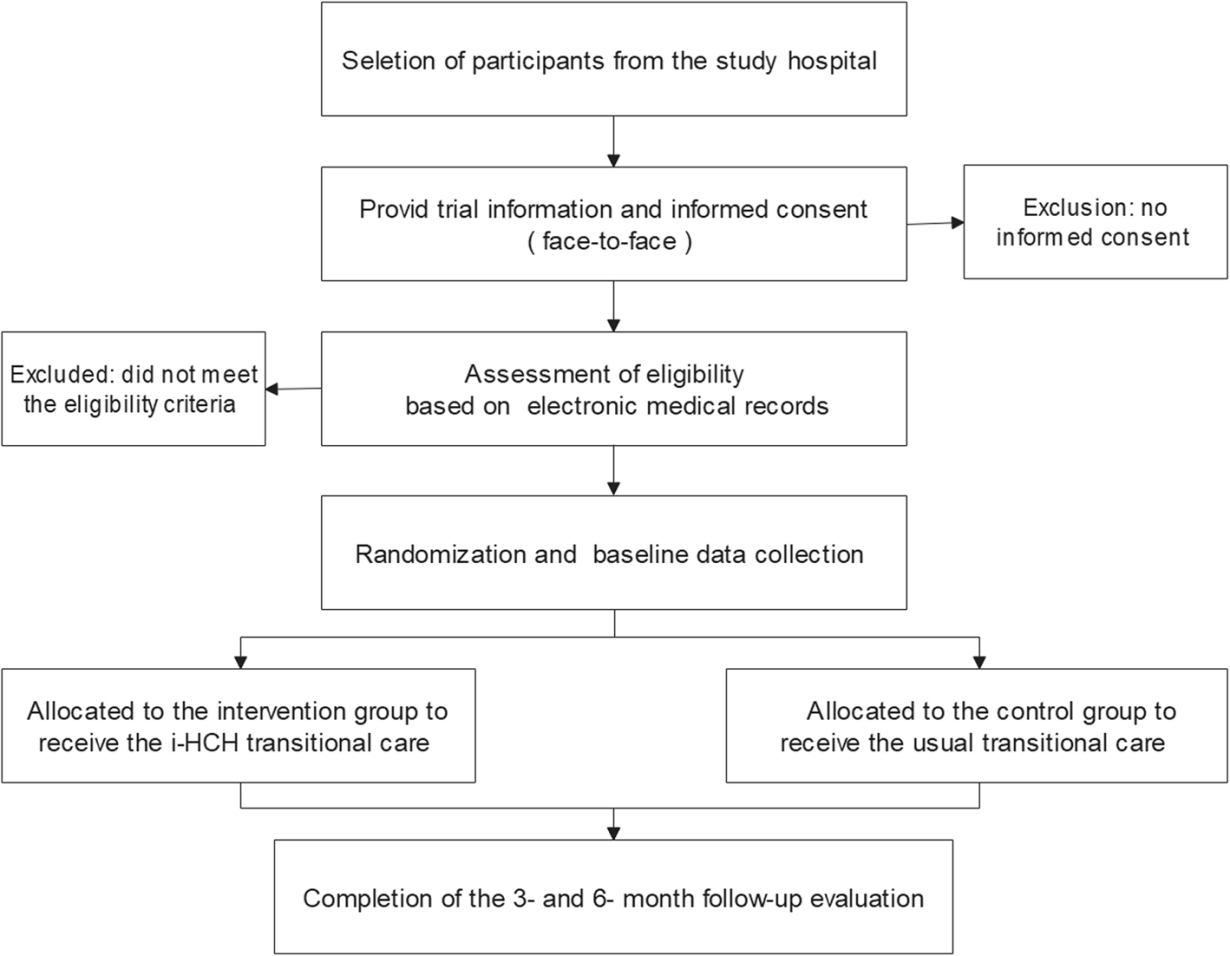
Study flowchart: participants' recruitment, randomization and follow-up

Participants' baseline data will be collected three days before discharge, and a questionnaire regarding demographics will be completed. Data collection will be conducted at 3 and 6 months after discharge. Patients will attend clinical appointments in the study hospital or designated community hospitals to complete clinical and laboratory assessments. Research assistants will upload the results to the diabetes management platform for secure collection. The research assistant will collect the questionnaires face-to-face during the outpatient visit. The patients' blood glucose data and hypoglycemia events will be supervised and consulted by CNs through the app or telephone. Readmission will be determined by referring to the participant's medical history in the general hospital. All data will be double-entered and checked for consistency. The study flowchart and participant data collection are depicted in Fig. 1 and Table 1.

**Table 1:**
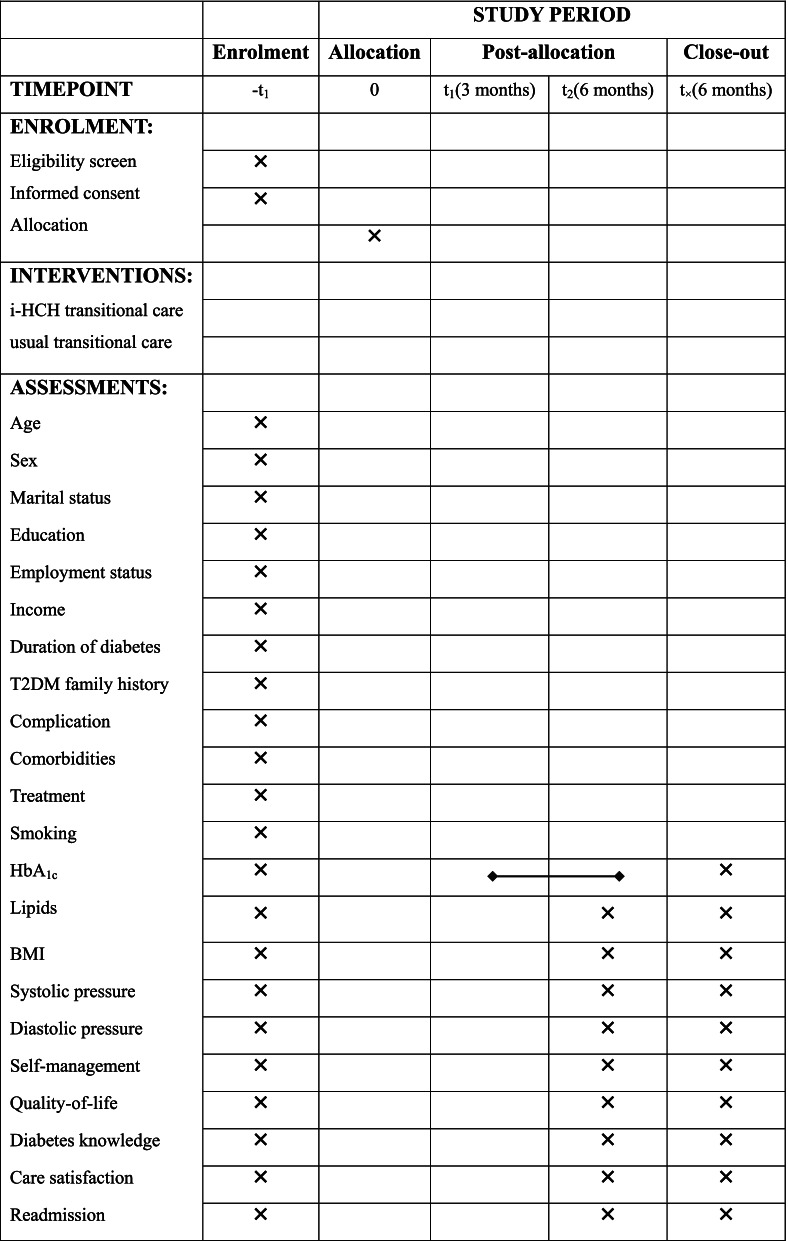
Summarize the schedule of enrolment, interventions, and assessments

### Description of the diabetes management system

The system comprises a patient app, a medical app and a management portal. In partnership with the mHealth Industrial Research Organization, the Department of Internet Information and Endocrinology at the General Hospital, and the community hospitals, we developed an app to address the integrated transitional care of T2DM patients. The app is user-friendly and modeled on a complete general hospital patient management platform that supports remote patient management.

The app can display various health records and the patients' physiological, biochemical, imaging examination, and hospitalization history data. With the patients' consent, the patients' family and transitional care term members can review relevant data on their mobile phones. The patient can manually enter the blood glucose value, blood pressure and body weight on the app, generating a dynamic curve. If these values exceed the target set by physicians, they will be marked in a significant color to alert the patient. Patients can check the frequency and dosage of oral medications and insulin in their recent prescriptions and record whether they have taken them through the app. The app can generate an individualized alarm clock that reminds patients to take their medications. In addition, the app supports recording daily food, physical activity and diabetes-related stress. The app automatically stratifies patients into three subgroups based on their blood glucose level and the frequency of health recordings and displays the subgroup on the medical app home page. The three patient subgroups include the following: warning patients with severe high or low blood glucose values (> 13.9 mmol/L or < 3.9 mmol/L); key patients with blood glucose values that exceed the target value or have no health recordings within a two-week time period; and general patients.

The app has a diabetes knowledge base from which patients and their families can read diabetes-related articles. The app is connected to the hospital outpatient system to conveniently allow patients to book outpatient appointments while updating the follow-up time and results. Its counseling function allows patients to actively communicate with the transitional care team when encountering adverse reactions or acute changes in their diabetes condition to seek professional help. Patient data will be regularly transmitted and uploaded to the management portal via the internet and subsequently presented in tabular or graphical formats for team members to oversee the patients' condition. We assessed clinician and patient demand for the diabetes app through a qualitative study and feasibility test (n = 17) to enhance the platform. This system will be utilized in this RCT.

### Study intervention

**Fig. 2:**
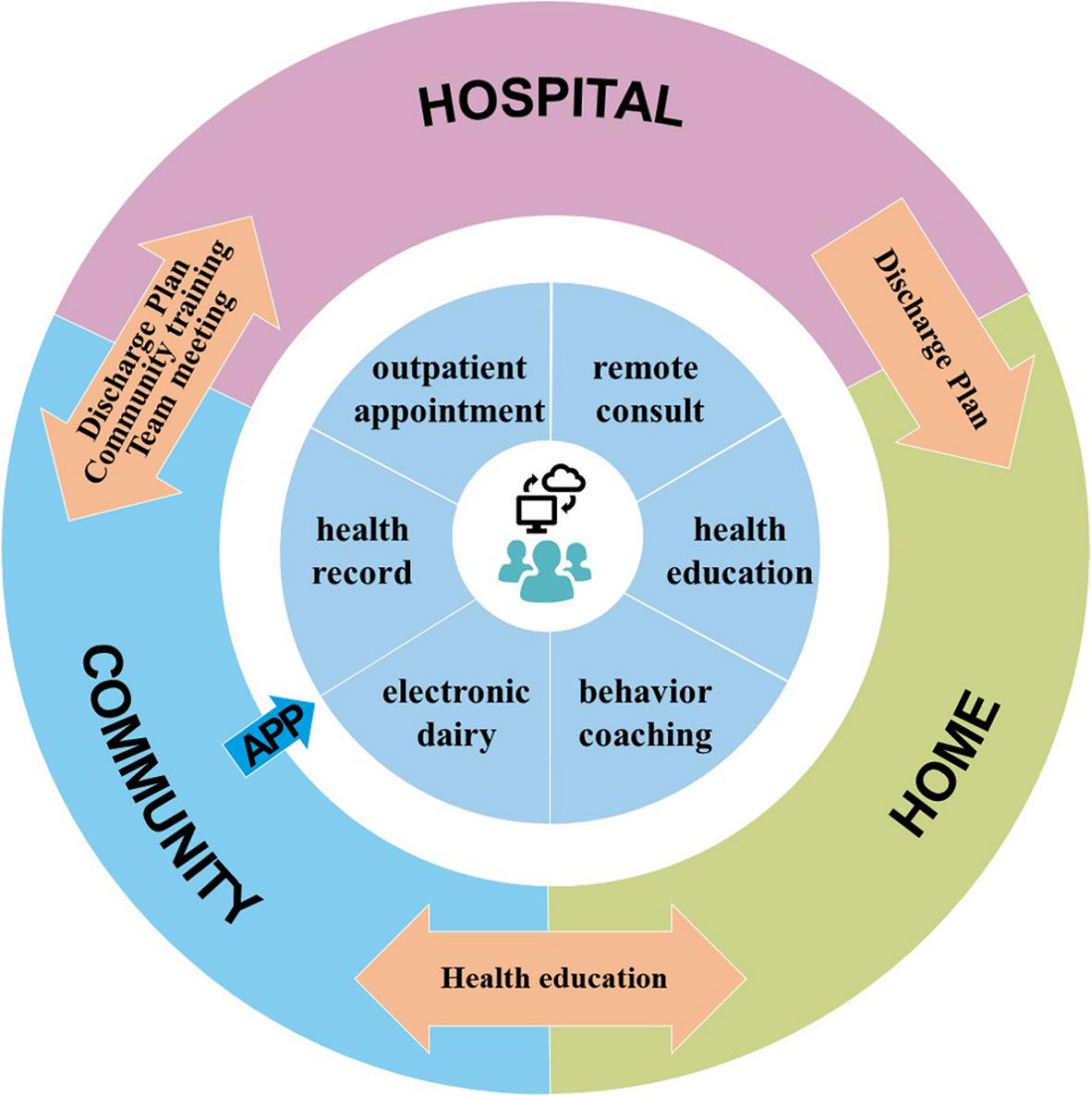
Summary of key components and features of the i-HCH program intervention for T2DM patients

Our intervention will be coordinated with a large general hospital, community hospitals and participants' families to provide integrated and continuous management to T2DM patients through the diabetes management system. Our intervention focuses on improving patient glycemic control and all essential aspects of self-management (blood glucose measurement, diet, exercise, medication adherence and diabetes-related knowledge). The i-HCH program intervention (Fig. 2) is designed to address the discontinuation of information and management between hospital and community care and enhance the role of family caregivers. The transitional care team will use the app to work directly with participants. Before the intervention, the team will undergo five days of training in the i-HCH program and app. The training aims to ensure that the care team has knowledge and interest in using the app to manage in-home participants in the long term. After the five days of training, the community team will continue to be trained every month on up-to-date clinical guidelines on diabetes knowledge and how to improve participants' self-management skills. CNs can consult with and refer to transitional care team members to obtain support to resolve the gap in care during the intervention.

Clinical nurses will be responsible for assessing individual symptoms, signs, and self-care behavior to make personalized discharge plans, including the patients' recent medical history, medication list, measurement frequency of blood glucose, and therapeutic target three days before discharge. Clinical nurses will encourage patients to select a family member who is willing and able to provide diabetes care at home and will install and demonstrate the app on participants' and family members' smartphones, giving them the motivation and skill to use the app. The clinical nurses will ensure that patients and their families are ready to be discharged and will share the discharge plan with them and the transitional care team.

CNs will remotely monitor the patients' health records at patient discharge through the app, including blood glucose, blood pressure, BMI, medication adherence, nutrition, physical activity, and emotion. The app will automatically provide alerts for abnormal indicator values and send texts on measurement target recommendations in real-time. CNs will implement individual interventions based on the risk stratification of patients by the app. They will use the app to send interactive messages once a month to patients, including messages setting evidence-based glycemic measurement goals, encouraging healthy behaviors and guiding family members to provide more additional assistance to in-home patients. CNs will identify the causes of poor blood glucose management in warning and key patients using the app counseling function and send tailor-made health recommendations. CNs will use the app to support and encourage patients to communicate with team members remotely. If the patient asks for a disease-related consultation, the CN or GP should handle it within 24 h. Patients have the option of refusing to accept the above information. Subsequently, CNs will continue to supervise the patient's blood glucose value and, if necessary, call or see the patient in their home.

Team members will transmit educational articles on the app weekly to patients and their families to help them learn about diabetes, such as hypoglycemia and signs of exacerbation. The pharmacist will provide professional pharmaceutical knowledge, such as skills on taking medication correctly, the importance of medication adherence and ways to adjust the insulin dose.

CNs will note app usage and self-care status and present health information for discussion in monthly team meetings. The intervention strategy will be adjusted accordingly to meet patients' needs when member consensus is achieved. Then, routine endocrinologist appointments will be performed in the third and sixth months after discharge. The app will send follow-up reminders to patients and provide information on the critical content and a preparation list two weeks before follow-up. Patients will be immediately recommended to schedule urgent appointments and will be referred to the general hospital with the responsible endocrinologist if they have unsafe glycemic levels or develop acute or chronic diabetic complications.

In the control group, participants will receive usual transitional care alone. Participants who achieve stable blood glucose are usually discharged with an in-home management plan drawn up by clinical nurses and physicians. Outpatients will attend regular clinic visits at the general hospital, with an interval of approximately three months. Participants can access health care from community hospitals in-between clinic visits if needed. The clinical nurses will perform a consultation to assess the outpatient's self-management condition once every three months and obtain blood glucose data that patients provide via a paper record or from a commercial glucose meter over the telephone or during a home visit; in addition, the clinical nurses will provide general advice and renew the diabetes management plan.

### Sample size

Our sample size estimation was performed using R Studio software based on HbA_1c_. A total of 126 participants from an endocrinologist in the general hospital are required to detect a mean difference in HbA_1c_ of 0.5% over six months between the intervention and control groups with a standard deviation of 1 to achieve 0.8 power and a two-sided alpha of 0.05. Allowing for a 10% attrition rate, the total participant number should be 138, or with 69 participants in each group.

### Statistical analysis

Data will be imported into SPSS 23.0 for further statistical analysis. Descriptive statistics, including the mean, standard deviation, and frequency, will summarize all participants' various factors. In order to determine whether the data met the planned statistical analysis hypothesis, the data were examined for distribution and collinearity before the analysis. The analysis will be based on an intention-to-treat (ITT), meaning that all eligible patient information was included in the analysis regardless of whether the intervention was completed. In the primary analysis, Student's t-tests or Mann–Whitney U tests will compare the differences in demographic and diabetes-related information in the two study groups at baseline and endpoint for continuous data or χ2 tests for proportions. The differential changes in repeated measurement data between groups will be assessed by repeated-measures ANOVA and the interaction effect (group × time) across three time points (baseline, three months, six months), aiming to evaluate the impact of the intervention over time on the primary study outcomes. The ITT analyses will also be used to impute missing study data. The analysis of secondary outcomes will be compared similarly to the primary analysis, except for the results of HbA_1c_ levels, where the remaining metrics will not be considered for repeated measurements based on the fact that they were measured ≤ 2 times. A p-value of less than 0.5 will be considered statistically significant.

## Discussion

As core members of primary care teams, CNs are becoming increasingly important in addressing the epidemic of chronic disease. CNs potentially have more excellent opportunities and the ability to provide continuing care than traditional clinical nurses, but lack professional training [8]. Communities have had insufficient resources to support such services on their own in China, and CNs have little capacity to meet certain patients' needs. Patients are usually seen in general hospitals, leading to overcrowding in hospitals, long waiting times, and overloading of transport and medical resources [37]. Community hospitals' strengths and services remain underused in chronic disease care. The local context has an important impact on the feasibility of forms of care [5]. Support for continuous and cooperative care with T2DM patients has not been achieved because of the lack of access to shared health information and work descriptions of different positions between local health care settings. In addition, patients' family members are extrinsic motivators to perform blood glucose management, and providing better family support and improving diabetes-related knowledge and attitudes will likely improve patients' health behaviors and outcomes [38, 39].

Specialist cooperation with primary care could reinforce glycemic control and prevent diabetes-related complications for T2DM patients. The i-HCH transitional care intervention was designed as a pragmatic RCT examining the feasibility of implementing continuing care in multiple settings via an app in the real-world setting of a hospital-community-home alliance. One of the key strengths of the study is refocusing on the cooperation and agreement between local hospitals and communities by designing a transitional care team workflow integration and emphasizing the complementary role of family members in disease management. The study addresses the discontinuity of information, contact and management in transitional care by clarifying team members' roles and work responsibilities and unifying network systems and devices. Ultimately, this study aims to optimize medical resource utilization to improve disease outcomes. The intervention has the potential to demonstrate significant improvements in diabetes-related biochemistry, self-management ability, quality of life and satisfaction of care for T2DM patients. If successful, it will positively impact T2DM patients, family members, health caregivers, and policymakers. This collaborative intervention program will continue to be applied in the hospital-community-home setting. If possible, we will analyze the cost-effectiveness of the intervention delivery and retention of app use at a later stage. Our study aims to verify the effectiveness of this program and directly contribute to constructing a research method for Chinese transitional care practice.

## Data Availability

Not applicable.

## Abbreviations

i-HCH: Integrated hospital-community-home
T2DM: Type 2 diabetes mellitus
mHealth: Mobile health
CNs: Community nurses
GPs: General practitioners
TC: Total cholesterol
TGs: Triglycerides
LDL: Low-density lipoprotein
HDL: High-density lipoprotein
BMI: Body mass index
